# Vascular invasion and lymph node metastasis mediate the effect of CA242 on prognosis in hilar cholangiocarcinoma patients after radical resection

**DOI:** 10.3389/fonc.2022.1071439

**Published:** 2022-12-12

**Authors:** Gang Heng, Benqi Huang, Yanbing Shen, Dan wang, Zhen Lan, Yuxuan Yao, Jianxin Zhang, Jiankun Jia, Chengcheng Zhang

**Affiliations:** ^1^ Department of Hepatobiliary Surgery, Southwest Hospital, Army Medical University, Chongqing, China; ^2^ Department of General Surgery, PLA Middle Military Command General Hospital, Wuhan, China; ^3^ Department of Quality Education, Jiangsu Vocational College of Electronics and Information, Huaian, China

**Keywords:** CA242, vascular invasion, lymph nodes metastasis, mediation analysis, prognosis

## Abstract

**Background:**

Carbohydrate antigen 242 has been clinically used as a diagnostic biomarker for pancreatic cancer. However, the prognostic role of CA242 in hilar cholangiocarcinoma (HCCA) has not been identified. Also, it remains unclear to what extents the vascular invasion and lymph node metastasis mediate the effect of serum CA242 on prognosis.

**Objective:**

This study aimed to investigate whether vascular invasion and lymph node metastasis mediate the relationship between CA242 levels and clinical prognosis in HCCA patients after radical resection.

**Methods:**

Data of 234 HCCA patients who accepted radical resection from March 2008 to December 2014 were analyzed. Vascular invasion and lymph node metastasis were assessed by postoperative pathological examinations. Mediation analysis was performed to study the potential causal relationship between CA242 and overall survival (OS) and relapse-free survival (RFS). Survival analysis was performed using the Kaplan-Meier method.

**Results:**

Among 234 HCCA patients, 104 patients (44.4%) with normal CA242 levels (≤ 20 IU/ml) had significantly better OS (p=0.004) and RFS (p=0.001) than those 130 patients (55.6%) with elevated CA242 levels (>20 IU/ml). The logistic analysis showed that elevated CA242 was an independent risk factor for vascular invasion (p=0.006) and lymph nodes metastasis (p=0.040). The causal mediation analysis indicated that the vascular invasion (p=0.012 for OS; p=0.036 for RFS) and lymph nodes metastasis (p=0.024 for OS; p=0.014 for RFS) played significant roles in mediating the effect of serum CA242 on OS and RFS.

**Conclusion:**

Serum elevated CA242 could be a novel marker for prognosis prediction in HCCA patients. Vascular invasion and lymph node metastasis mediated the relationship between CA242 and clinical prognosis.

## Introduction

Hilar cholangiocarcinoma (HCCA) primarily originates from hilar bile duct, accounting for about 50% of cholangiocarcinoma (1). Radical resection is the only potential cure to this disease, while the 5-year overall survival (OS) rate after resection ranges from 15% to 40% (2, 3). Previous works demonstrate that tumor differentiation, vascular invasion, lymph node metastasis and histological grade are the prognostic factors (4, 5). However, these factors mostly rely on postoperative pathological examination which cannot provide oncologists with prospective advice in personalized treatment adjustment.

Carbohydrate antigen 242 (CA242) is a sialylated glycosphingolipid antigen that is clinically used for the diagnosis and prognosis prediction of malignant tumors of the digestive tract. Elevated CA242 has been found in pancreatic cancer cells, and the expression level of which is correlated with the development of pancreatic cancer and other gastric intestinal tract cancers (6). However, as a tumor marker with good sensitivity and specificity in cancer, the prognostic value of preoperative serum CA242 is rarely reported in HCCA. Besides, according to clinical practice, serum CA242 might be an indirect factor affecting prognosis, and the mediators between CA242 and clinical prognosis are unknown. As the major components of TNM staging, vascular invasion (hepatic artery and portal vein invasion) and lymph node metastasis are direct prognostic factors for HCCA (7, 8). Whether the effect of serum CA242 on clinical prognosis is mediated by the vascular invasion and lymph node metastasis has not been studied.

In this study, we aim to assess whether elevated CA242 is an independent risk factor to predict clinical prognosis of HCCA patients after radical resection, and how the vascular invasion and lymph node metastasis interact with the effect of serum CA242 on clinical prognosis.

## Methods

### Study population

A total of 234 HCCA patients admitted to the Southwest Hospital of Army Medical University from March 2008 to December 2014, were included in the study. The study was approved by the Ethical Committee of Southwest Hospital, the first affiliated Hospital of Army Medical University. Written informed consent was obtained from all patients.

### Data collection

The inclusion criteria of this study included: patients with pathological confirmation of HCCA; patients whose preoperative serum CA242 level was evaluated; patients received R0 resection.

The demographic, biochemical and surgical characteristics of each HCCA patient were collected, including age, gender, weight, hypertension, diabetes, hepatolithiasis, tumor size, lymph node metastasis, tumor differentiation, CA242, CA19-9, CEA, CA125, total bilirubin, albumin, Bismuth-Corletta classification and vascular invasion. Vascular invasion in this study was defined as macrovascular invasion (portal vein or hepatic artery in the hilar region), which was verified by intraoperative observation and postoperative pathological examination.

All patients in this study accepted R0 resection with histologically negative margins. The proximal end of the bile duct greater than 5 mm from the edge of the tumor, and the distal end at the upper edge of the pancreas were resected in the operation. The entire caudate lobe and surrounding liver parenchyma greater than 15 mm surrounding the bile duct axis were removed. For patients with Bismuth-Corlette III or IV HCCA, hemihepatectomy or extended hemihepatectomy and caudate lobectomy were conducted. Lymph nodes of groups 8, 12 and 13 were conventionally dissected.

### Follow-up

These patients who accepted R0 resection did not routinely accept adjuvant therapy after postoperation. All individuals were closely followed up in outpatient clinic at Southwest Hospital after discharge. Basic measurement including liver function and blood routine examination, examination of serum tumor markers and imaging were performed. Overall survival (OS) was calculated from the day of surgery.

### Mediation analysis

**Figure 1:**
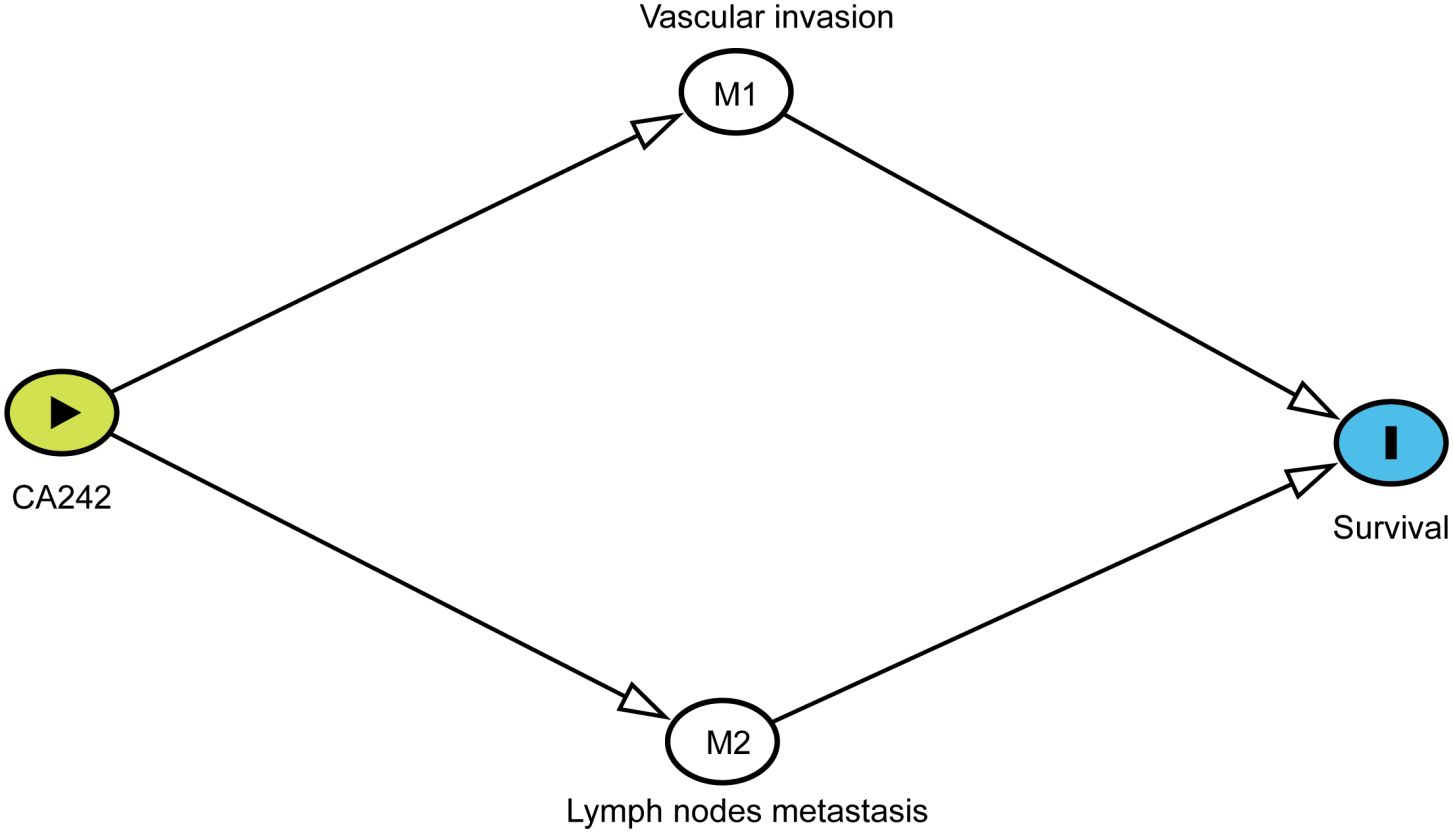
The DAG (Directed Acycling Graph) showing the relationship between CA242, vascular invasion, lymph node metastasis and prognostic survival.

Causal mediation analysis was used to study the effects mediated by vascular invasion and lymph node metastasis between CA242 and overall survival (OS) or recurrence-free survival (RFS). To perform mediation analysis, Chi-square tests and multivariate logistic regression analysis were performed to characterize the relationship between elevated CA242 and vascular invasion and lymph nodes metastasis. Cox regression model was used to investigate the association between vascular invasion/lymph node metastasis and clinical prognosis. Then, the DAG (Directed Acycling Graph) was created in DAGitty according to the previous analysis (**Figure 1**) (9). The proportion mediated by vascular invasion/lymph node metastasis between CA242 and OS/RFS was computed using bootstrap methods (resamples = 1000).

### Statistical analysis

Continuous variables are presented as the mean and standard deviation (SD) or median and interquartile range (IQR). Categorical variables are presented as amounts and percentages. Comparisons between clinicopathological factors were made using Student’s t test or analysis of variance or Kruskal-Wallis test for continuous variables and χ2 test for categorical variables.

Cox regression was used for univariate and multivariate analyses, and the mediating variables were removed in the adjusted Cox regression model. Confounding variables were included in the Cox regression model. Kaplan—Meier curves were used to estimate OS and RFS.

All statistical analyses were performed in STATA (version 14). Significant variables in univariate regression will be included in multivariate analysis, and p<0.05 was considered statistically significant.

## Results

### Patient characteristics

In this study, a cohort of 234 patients HCCA were included. There were 129 men and 105 women in the cohort, with a mean age of 57.9 years. For all patients, the median follow-up was 24.7 months (range, 1-136 months). The median OS and RFS time were 21 months and 16 months, respectively. The details of demographic and clincopathological data between normal CA242 (CA242 <= 20 IU/ml) and elevated CA242 (CA242 > 20 IU/ml) were given in **Table 1**. The serum levels of CA199 and CA125 were significantly higher in the elevated CA242 group (p=0.001 and p=0.003, respectively). In addition, there were significantly more patients in the elevated CA242 group (38.3% vs 20.2%, p=0.003) accepted PTCD before operation in comparing with normal CA242 group. The proportions of patients with vascular invasion (hepatic artery invasion or portal vein invasion or both) and positive lymph nodes in elevated CA242 group were also higher than normal CA242 group (48.5% vs 26.0%, p=0.001; 44.6% vs 30.8%, p=0.021).

**Table 1:** Baseline characteristics between two groups stratified by CA242 level.

Variable	CA242 <= 20 IU/ml (N = 104)	CA242 > 20 IU/ml (N = 130)	p value
*Epidemiologic features*			
Age (years), mean ± SD	57.6 ± 10.0	58.3 ± 10.6	0.591
Gender/male, n (%)	53 (51.0%)	76 (58.5%)	0.252
Weight (kg)	59.0 ± 8.8	57.0 ± 9.2	0.119
Hepatolithiasis, n (%)	8 (7.7%)	12 (9.5%)	0.637
Diabetes, n (%)	9 (8.7%)	14 (11.0%)	0.550
Hypertension, n (%)	20 (19.2%)	13 (10.2%)	0.052
*Laboratory tests*			
WBC (x10^9^/L)	9.8 ± 28.7	7.7 ± 5.8	0.446
Platelet (x10^9^/L)	234.8 ± 90.8	250.9 ± 93.0	0.194
ALT (IU/L)	133.5 ± 128.1	117.6 ± 115.8	0.462
AST (IU/L)	115.9 ± 102.9	108.5 ± 102.6	0.591
Total bilirubin (µmol/L)	172.7 ± 128.0	181.0 ± 115.4	0.608
Direct bilirubin (µmol/L)	88.2 ± 65.7	102.3 ± 72.3	0.129
Albumin (g/L)	37.2 ± 4.0	36.1 ± 5.5	0.088
AKP (U/L)	513.1 ± 395.6	475.3 ± 321.1	0.431
γ-GGT (U/L)	682.3 ± 701.5	493.8 ± 398.0	0.013
PT (s)	11.8 ± 9.0	12.2 ± 6.9	0.712
CEA (µg/L)	5.0 ± 13.1	6.9 ± 19.9	0.418
CA199 (kU/L)	162.9 ± 209.0	716.6 ± 1070.1	0.001
CA125 (U/mL)	15.4 ± 11.7	34.5 ± 59.6	0.003
PTCD, n (%)	21 (20.2%)	49 (38.3%)	0.003
*Clinico-Pathological features*			
Liver cirrhosis, n (%)	8 (7.7%)	8 (6.2%)	0.655
Bismuth-Corletta classification, n (%)			
Type III and IV	62 (60.2%)	77 (59.7%)	0.938
Hepatic artery invasion, n (%)	16 (15.4%)	30 (23.1%)	0.141
Portal vein invasion, n (%)	24 (23.1%)	52 (40.0%)	0.006
Vascular invasion, n (%)	27 (26.0%)	63 (48.5%)	0.001
Positive lymph nodes, n (%)	32 (30.8%)	58 (44.6%)	0.021
Poor differentiation, n (%)	20 (20.2%)	25 (21.0%)	0.884
Number of positive lymph nodes, (n)	1.1 ± 2.4	1.2 ± 1.5	0.634
Endoscopic vascular invasion, n (%)	11 (18.3%)	16 (20.8%)	0.721
Endoscopic nerve invasion, n (%)	45 (62.5%)	40 (43.5%)	0.016
AJCC TNM stage ≥ III, n (%)	50 (48.1%)	93 (71.5%)	0.001
Tumor size > 2.5 cm, n (%)	51 (49.0%)	88 (67.7%)	0.004
Major complication, n (%) (Clavien-Dindo ≥ III)	11 (10.6%)	13 (10.0%)	0.885

### Serum elevated CA242 is an independent risk factor for vascular invasion and lymph node metastasis

Univariable and multivariable analysis were conducted to investigate the relationship between serum CA242 and vascular invasion. The results were shown in **Table 2**, the elevated CA242 (OR=2.619, p=0.006) and Type III and IV Bismuth-Corletta classification (OR=2.816, P=0.001) were independent risk factors for vascular invasion, while other tumor markers like CA199, CEA and CA125 did not show significant association with vascular invasion.

**Table 2:** Univariable and multivariable analysis of risk factors associated with vascular invasion.

Factors	Univariable analysis		Multivariable analysis	
	OR (95% CI)	p value	OR (95% CI)	p value
Age > 60	0.808 (0.477 - 1.371)	0.431	0.702 (0.380 – 1.296)	0.258
Male	1.380 (0.811 - 2.348)	0.236		
Hepatolithiasis	0.864 (0.341 – 2.199)	0.766		
Liver cirrhosis	2.854 (1.036 – 7.849)	0.042		
Diabetes	0.686 (0.278 – 1.700)	0.425		
Total bilirubin > 21 (µmol/L)	1.977 (0.717 – 5.423)	0.196		
Preoperative PTCD	1.674 (0.948 – 2.957)	0.076	1.442 (0.742 – 2.805)	0.281
Albumin < 30 (g/L)	0.882 (0.230 – 2.604)	0.826		
ALT > 40 (IU/L)	1.000 (0.497 – 2.008)	1.000		
CA199 > 35 (U/mL)	2.556 (1.070 – 6.083)	0.034	1.773 (0.654 – 4.801)	0.260
CEA > 5 (µg/L)	1.370 (0.701 – 2.679)	0.361		
CA125 > 35 (U/mL)	1.044 (0.437 – 2.505)	0.924		
CA242 > 20 (IU/mL)	3.240 (1.789 – 5.871)	<0.001	2.619 (1.324 - 5.181)	0.006
Bismuth-Corletta classification (≥ III)	2.389 (1.359 – 4.197)	0.002	2.816 (1.495 – 5.307)	0.001
Tumor size > 2.5 (cm)	1.523 (0.885 – 2.619)	0.130		
Poor tumor differentiation	1.756 (0.911 – 3.387)	0.094		

Similarly, the relationship between CA242 and lymph node metastasis was studied using univariable and multivariable analysis (**Table 3**). It was shown that the serum elevated CA242 was also significantly associated with lymph node metastasis (OR=1.974, p=0.040).

**Table 3:** Univariable and multivariable analysis of risk factors associated with lymph nodes metastasis.

Factors	Univariable analysis		Multivariable analysis	
	OR (95% CI)	p value	OR (95% CI)	p value
Age > 60	1.040 (0.613 - 1.763)	0.886	0.851 (0.456 – 1.590)	0.613
Male	1.204 (0.708 - 2.049)	0.494		
Hepatolithiasis	0.397 (0.134 – 1.183)	0.140		
Liver cirrhosis	0.765 (0.264 – 2.221)	0.634		
Diabetes	0.529 (0.207 – 1.362)	0.194		
Total bilirubin > 21 (µmol/L)	0.611 (0.249 – 1.496)	0.290		
Preoperative PTCD	1.689 (0.953 – 2.992)	0.073	1.538 (0.772 – 3.064)	0.221
Albumin < 30 (g/L)	2.195 (0.766 – 6.282)	0.150		
ALT > 40 (IU/L)	1.215 (0.589 – 2.503)	0.602		
CA199 > 35 (U/mL)	1.079 (0.501 – 2.320)	0.847		
CEA > 5 (µg/L)	1.405 (0.720 – 2.740)	0.322		
CA125 > 35 (U/mL)	2.543 (1.060 – 6.093)	0.036	1.720 (0.611 – 4.841)	0.304
CA242 > 20 (IU/mL)	2.350 (1.314 – 4.202)	0.004	1.974 (1.030 - 3.783)	0.040
Bismuth-Corletta classification (≥ III)	1.043 (0.609 – 1.786)	0.880		
Tumor size > 2.5 (cm)	1.582 (0.918 – 2.726)	0.099	1.601 (0.836 – 3.063)	0.155
Poor tumor differentiation	0.543 (0.269 – 1.098)	0.090	0.589 (0.269 – 1.293)	0.187

### Vascular invasion and lymph node metastasis mediate the effect of serum CA242 on clinical prognosis

Accordingly, vascular invasion and lymph node metastasis were independent risk factors for clinical prognosis in HCCA patients. In this study, we also verified this relationship with Cox regression model. It was shown in **Table 4** that vascular invasion (HR=1.536, p=0.014) and lymph node metastasis (HR=1.739, p=0.001) were significantly associated with OS. For RFS, vascular invasion (HR=1.427, p=0.034) and lymph node metastasis (HR=1.901, p=0.042) were also independent risk factors (**Table 5**). Besides, as shown in the unadjusted model for OS and RFS (**Tables 4**, **5**), serum CA242 was not an independent prognostic factor (For OS: HR=1.258, p=0.209; For RFS: HR=1.296, p=0.161).

**Table 4:** Multivariable analysis of risk factors associated with overall survival.

Factors	Unadjusted model		Adjusted model^a^	
	HR (95% CI)	p value	HR (95% CI)	p value
Age > 60	1.101 (0.801 - 1.513)	0.551	1.090 (0.794 – 1.498)	0.593
Total bilirubin > 21 (µmol/L)	1.237 (0.645 – 2.374)	0.522	1.119 (0.588 – 2.128)	0.733
Albumin < 30 (g/L)	1.759 (0.835 –3.703)	0.137	2.256 (1.082 –4.705)	0.030
CA242 > 20 (IU/mL)	1.258 (0.879 –1.799)	0.209	1.518 (1.083 - 2.128)	0.015
Preoperative PTCD	0.973 (0.678 – 1.398)	0.886	1.045 (0.727 - 1.502)	0.810
Bismuth-Corletta classification (≥ III)	0.878 (0.635 –1.213)	0.430	0.891 (0.645 – 1.232)	0.487
Tumor size > 2.5 (cm)	1.062 (0.750 –1.502)	0.736	1.196 (0.848 –1.688)	0.307
Poor tumor differentiation	1.842 (1.247 – 2.723)	0.002	1.824 (1.241 –2.682)	0.002
Vascular invasion	1.536 (1.090 –2.164)	0.014		
Lymph nodes metastasis	1.739 (1.238 –2.443)	0.001		

a Mediators (vascular invasion and lymph nodes metastasis) were not included in the adjusted model.

**Table 5:** Multivariable analysis of risk factors associated with relapse-free survival.

Factors	Unadjusted model		Adjusted model^a^	
	HR (95% CI)	p value	HR (95% CI)	p value
Age > 60	0.877 (0.633 - 1.216)	0.431	0.855 (0.618 –1.183)	0.345
Total bilirubin > 21 (µmol/L)	1.565 (0.791 – 3.096)	0.199	1.331 (0.680 –2.606)	0.404
Albumin < 30 (g/L)	1.883 (0.849 – 4.178)	0.119	2.376 (1.081 –5.221)	0.031
CA242 > 20 (IU/mL)	1.296 (0.902 – 1.862)	0.161	1.568 (1.115 - 2.205)	0.010
Preoperative PTCD	0.865 (0.599 – 1.248)	0.438	0.841 (0.653 –1.357)	0.747
Bismuth-Corletta classification (≥ III)	0.966 (0.698 – 1.338)	0.836	0.964 (0.696 – 1.336)	0.828
Tumor size > 2.5 (cm)	1.089 (0.770 –1.542)	0.628	1.233 (0.876 – 1.737)	0.230
Poor tumor differentiation	1.572 (1.034 –2.390)	0.034	1.525 (1.003 – 2.320)	0.049
Vascular invasion	1.427 (1.012 –2.014)	0.034		
Lymph nodes metastasis	1.901 (1.346 –2.683)	0.042		

a Mediators (vascular invasion and lymph nodes metastasis) were not included in the adjusted model.

According to previous analysis, we drew the DAG to indicate the potential relationship of these critical variables for HCCA patients. Each relationship in the DAG was verified. As shown in **Figure 1**, vascular invasion and lymph node metastasis were direct variables affecting the survival. The effect of CA242 played on survival was mediated by vascular invasion and lymph node metastasis. Therefore, vascular invasion and lymph node metastasis were two critical mediators between CA242 and clinical prognosis.

**Figure 2:**
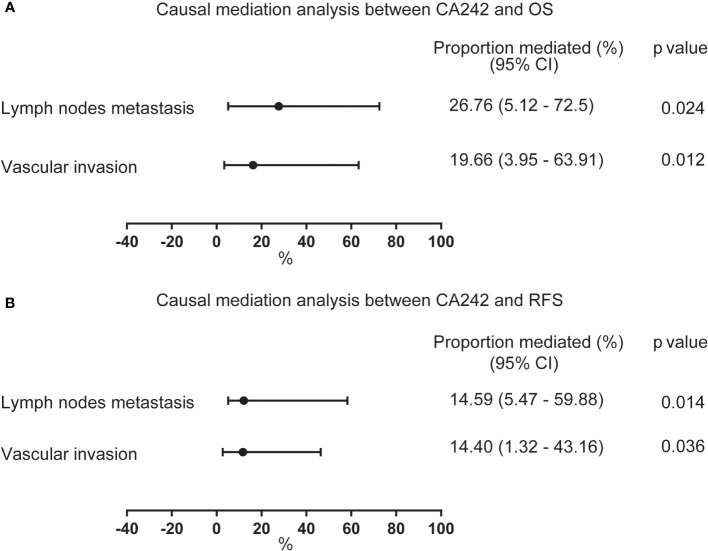
The forest plot showing the proportion mediated by vascular invasion and lymph node metastasis between CA242 and OS/RFS. **(A)** The average proportion mediated by lymph node metastasis and vascular invasion between serum elevated CA242 levels and OS. **(B)** The average proportion mediated by lymph node metastasis and vascular invasion between serum elevated CA242 levels and RFS.

Afterwards, we conducted a causal mediation analysis between CA242 and OS/RFS. As shown in **Figure 2**, the average proportion mediated by lymph node metastasis and vascular invasion between CA242 and OS were 26.76% (CI: 5.12%-72.5%, p=0.024) and 19.66% (CI: 3.95%-63.91%, p=0.012), respectively. For RFS, the average proportion mediated by lymph node metastasis and vascular invasion were 14.59% (CI: 5.47%-59.88%, p=0.014) and 14.40% (CI: 1.32%-43.16%, p=0.036), respectively.

### Serum CA242 as an independent factor for prognosis without mediators

The results of the conventional model including vascular invasion and lymph node metastasis these two mediators showed that the serum CA242 was not an independent risk factor for OS and RFS (**Tables 4**, **5**). However, in the adjusted model without those mediators, it was found that CA242 level (HR=1.518, p=0.015) was significantly associated with the postoperative OS. Similarly, the adjusted model without mediators indicted serum CA242 level as an independent risk factor for RFS (HR=1.568, p=0.010) (**Table 5**).

**Figure 3:**
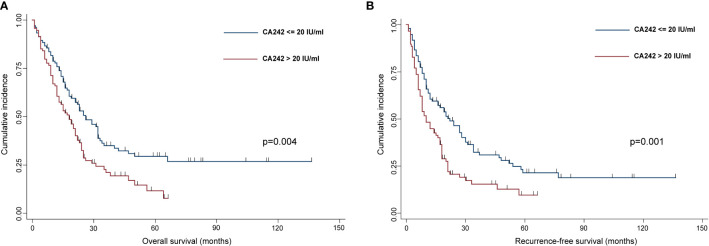
The Kaplan-Meier curves indicated the OS **(A)** and RFS **(B)** in HCCA patients.

Then, we did a Kaplan-Meier curves to estimate OS and RFS between normal serum CA242 (≤20 IU/ml) group and elevated CA242 (>20 IU/ml) group, and found that patients in normal CA242 group showed a better prognosis than patients in the elevated CA242 group (p=0.004 for OS, p=0.001 for RFS) (**Figure 3**).

## Discussion

HCCA, as a malignant tumor with highly aggressive behavior, has been intractable for hepatobiliary oncologists. Plenty of efforts have been put forward to evaluate the prognosis of HCCA, such as TMN staging, tumor location, papillary histology and primary sclerosing cholangitis (10). In this study, we found that serum CA242, a sialylated carbohydrate antigen used to diagnose and assess the prognosis of gastrointestinal cancer, could be an independent risk factor for clinical prognosis (11). Besides, we verified that the effect of serum CA242 on prognosis was mediated by vascular invasion and lymph node metastasis. As we know, it is the first study to investigate this relationship.

In previous report, vascular invasion, including hepatic artery and portal vein invasion, and lymph node metastasis played critical roles in assessing the surgical resectability, excision extension and clinical prognosis (12). However, the accuracy of preoperative imaging technologies like CT or MRI to evaluate the situation of major vessels and lymph nodes was still limited, which was largely determined by pathological examination postoperatively (13). Therefore, finding another approach to help diagnose the vascular invasion and lymph node metastasis has been of great importance. To our knowledge, serum tumor markers such as CEA, CA199 and CA125, have not been studied to predict the vascular invasion and lymph node metastasis in HCCA (14–16). In this study, we found that serum elevated CA242 level was significantly associated with the vascular invasion and lymph node metastasis in HCCA patients using logistic regression model, indicating serum CA242 could be used to predict vascular invasion and lymph node metastasis. This might help hepatobiliary surgeon to assess tumor resectability, excision extension and clinical prognosis before operation.

As a common tumor antigen, serum CA242 was produced and secreted into peripheral blood by cancer cells, which was investigated by biochemical and immunohistochemical studies (17). Lots of researches proved that high serum CA242 level is the independent risk factor for the poor prognosis of the radical pancreatic ductal adenocarcinoma (PDAC) (18–20). Also, the sensitivity and specificity of serum CA242 in the diagnosis of pancreatic cancer were 0.703 and 0.865 (18). In colorectal cancer, CA242 had strong consistencies in the diagnosis and prognosis for predicting long-time survival (21). Besides, Tao et al. indicated that serum CA242 was a better marker in distinguishing intrahepatic cholangiocarcinoma (ICCA) from hepatocellular carcinoma (HCC) (22). In pancreatic cancer patients, Ye Chen et al. found that patients with tumor diameter >4 cm exhibited higher serum CA242 levels than those with tumor diameter ≤4 cm (23). This was also found in our study for HCCA that patients with elevated CA242 experienced larger tumor size than those with normal CA242 levels, indicating that serum CA242 level might be significantly associated with tumor size. As reported, tumor size has long been studied as an independent risk factor for local invasion and metastasis in various cancers (24–26). Therefore, this might be the reason that elevated serum CA242 was associated with vascular invasion and lymph node metastasis.

DAG, is a theory-driven method to screen appropriate independent variables to enter the statistical model, building a causal network based on the theoretical causality (27). DAG has been widely used to analyze the economic and epidemic questions, while it is not used in establishing the relationship between different variables in HCCA patients. According to the clinical practice and AJCC staging system, vascular invasion and lymph node metastasis were direct variables affecting the prognosis of HCCA patients after R0 resection. After that, we validated that serum CA242 was an independent risk factor for vascular invasion and lymph node metastasis. Thus, vascular invasion and lymph node metastasis were two critical mediators between serum CA242 and clinical prognosis in the DAG. Each relationship in the DAG was verified in the **Tables 2**-**5**. Other variables like tumor volume, tumor differentiation and Bismuth-Corlette classification in the DAG network were considered as confounding variables. For hepatobiliary operation, the preoperative liver function status played a vital role in determining the excision extension, postoperative complication and prognosis, so some key variables like albumin and bilirubin were also considered as confounding variables (28–30). In the conventional regression models, all clinically or statistically significant variables, no matter mediator or confounders, were included in the models to identify the risk factors for prognosis (14, 31). However, this might result in a great bias due to the potential multicollinearity (32). In this study, a DAG was introduced in this study to screen the potential mediators and confounders, and then the mediators were excluded from the multivariable Cox regression model (33). Therefore, the conclusion of serum CA242 could serve as an independent factor for prognosis drew form the adjusted regression model, was relatively reliable.

However, there are some apparent limitations in this study. First, only the HCCA patients accepting radical resection were included. The conclusion might not applicable to all HCCA patients, as those who accepted palliative resection or conservative treatment were not analyzed. Second, the number of patients enrolled in this study was relatively small and came from a single center, causing a lack of regional applicability in the results. Third, all bias related to retrospective study cannot be avoided completely, although the causal mediation analysis and DAG were used.

## Conclusion

To summarize, we found vascular invasion and lymph node metastasis were two significant mediators between serum CA242 and prognosis in HCCA patients after radical resection, and serum CA242 could be a novel marker to predict clinical prognosis.

## Data availability statement

The original contributions presented in the study are included in the article/supplementary material. Further inquiries can be directed to the corresponding authors.

## Ethics statement

The studies involving human participants were reviewed and approved by the Ethical Committee of Southwest Hospital, The First Affiliated Hospital of Army Medical University. The patients/participants provided their written informed consent to participate in this study.

## Author contributions

CZ, JJ, and JZ designed the study. GH and BH performed the research and wrote the article. YS, DW, ZL and YY collected the data and conducted primary statistical analysis. All authors contributed to the article and approved the submitted version.
